# Maintenance of Energy Homeostasis during Calorically Restricted Ketogenic Diet and Fasting-MR-Spectroscopic Insights from the ERGO2 Trial

**DOI:** 10.3390/cancers12123549

**Published:** 2020-11-27

**Authors:** Katharina J. Wenger, Marlies Wagner, Patrick N. Harter, Kea Franz, Jörg Bojunga, Emmanouil Fokas, Detlef Imhoff, Claus Rödel, Johannes Rieger, Elke Hattingen, Joachim P. Steinbach, Ulrich Pilatus, Martin Voss

**Affiliations:** 1 Institute of Neuroradiology, University Hospital Frankfurt, Goethe University, 60528 Frankfurt am Main, Germany; marlies.wagner@kgu.de (M.W.); elke.hattingen@kgu.de (E.H.); u.pilatus@em.uni-frankfurt.de (U.P.); 2 University Cancer Center Frankfurt (UCT), University Hospital Frankfurt, Goethe University, 60590 Frankfurt am Main, Germany; patrick.harter@kgu.de (P.N.H.); kea.franz@kgu.de (K.F.); emmanouil.fokas@kgu.de (E.F.); detlef.imhoff@kgu.de (D.I.); clausmichael.roedel@kgu.de (C.R.); joachim.steinbach@kgu.de (J.P.S.); martin.voss@kgu.de (M.V.); 3 German Cancer Consortium (DKTK) Partner Site Frankfurt/Mainz, 60590 Frankfurt am Main, Germany; 4 Frankfurt Cancer Institute (FCI), 60590 Frankfurt am Main, Germany; Joerg.Bojunga@kgu.de; 5 Neurological Institute (Edinger-Institute), University Hospital Frankfurt, Goethe University, 60528 Frankfurt am Main, Germany; 6 Department of Neurosurgery, University Hospital Frankfurt, Goethe University, 60528 Frankfurt am Main, Germany; 7 Department of Medicine, University Hospital Frankfurt, Goethe University, 60590 Frankfurt am Main, Germany; 8 Department of Radiotherapy and Oncology, University Hospital Frankfurt, Goethe University, 60590 Frankfurt am Main, Germany; 9 Dr. Senckenberg Institute of Neurooncology, University Hospital Frankfurt, Goethe University, 60528 Frankfurt am Main, Germany; j.rieger@uni-tuebingen.de; 10 Interdisciplinary Division of Neuro-Oncology, University Hospital Tübingen, 72076 Tübingen, Germany

**Keywords:** glioblastoma, ketogenic diet, fasting, MR-spectroscopy, ketone body, ATP

## Abstract

**Simple Summary:**

The glioblastoma is a highly malignant brain tumor with very limited treatment options up to date. Metabolism of this tumor is highly dependent on glucose uptake. It is believed that glioblastoma cells cannot metabolize ketone bodies, which are found in the blood during periods of fasting or ketogenic dieting. According to this hypothesis, dieting could lead to cancer cell starvation. The ERGO2 (Ernaehrungsumstellung bei Patienten mit Rezidiv eines Glioblastoms) MR-spectroscopic imaging subtrial was designed to investigate tumor metabolism in patients randomized to calorically restricted ketogenic diet/intermittent fasting versus standard diet. The non-invasive investigation of tumor metabolism is of high clinical interest.

**Abstract:**

*Background:* The ERGO2 (Ernaehrungsumstellung bei Patienten mit Rezidiv eines Glioblastoms) MR-spectroscopic imaging (MRSI) subtrial investigated metabolism in patients randomized to calorically restricted ketogenic diet/intermittent fasting (crKD-IF) versus standard diet (SD) in addition to re-irradiation (RT) for recurrent malignant glioma. Intracerebral concentrations of ketone bodies (KB), intracellular pH (pH_i_), and adenosine triphosphate (ATP) were non-invasively determined. *Methods:* 50 patients were randomized (1:1): Group A keeping a crKD-IF for nine days, and Group B a SD. RT was performed on day 4–8. Twenty-three patients received an extended MRSI-protocol (^1^H decoupled ^31^P MRSI with 3D chemical shift imaging (CSI) and 2D ^1^H point-resolved spectroscopy (PRESS)) at a 3T scanner at baseline and on day 6. Voxels were selected from the area of recurrent tumor and contralateral hemisphere. Spectra were analyzed with LCModel, adding simulated signals of 3-hydroxybutyrate (βOHB), acetone (Acn) and acetoacetate (AcAc) to the standard basis set. *Results:* Acn was the only reliably MRSI-detectable KB within tumor tissue and/or normal appearing white matter (NAWM). It was detected in 4/11 patients in Group A and in 0/8 patients in Group B. MRSI results showed no significant depletion of ATP in tumor tissue of patients at day 6 during crKD-IF, even though there were a significant difference in ketone serum levels between Group A and B at day 6 and a decline in fasting glucose in Group A from baseline to day 6. The tumor specific alkaline pH_i_ was maintained. *Conclusions:* Our metabolic findings suggest that tumor cells maintain energy homeostasis even with reduced serum glucose levels and may generate additional ATP through other sources.

## 1. Introduction

Despite multimodal treatment options including surgical resection [1], radiotherapy, and/or chemotherapy [2] as well as tumor treating fields [3], glioblastoma (GBM) continues to carry a poor prognosis. Only 15–20% of patients survive longer than three years [4]. A standard second line treatment has not yet been established. According to current guidelines, treatment at recurrence can consist of re-resection, chemotherapy or re-irradiation [5]. With regard to chemotherapy, alkylating substances (re-challenge with either temozolomide or nitrosoureas such as lomustine) are the most commonly used treatment options [6]. Re-irradiation shows low toxicity even for patients with large tumor volumes [7], but median progression free survival (PFS) is restricted to approximately 5 months [8].

According to Otto Warburg’s theory, malignant cells display high rates of glycolysis and lactate production, even in the presence of adequate oxygen [9,10]. Activated Protein kinase B (PKB/Akt) has been shown to stimulate glucose consumption in malignant cells without affecting the rate of oxidative phosphorylation, rendering cancer cells dependent on aerobic glycolysis for continued growth and survival [11]. To a large extend, increased glucose metabolism in GBM can be plausibly explained through activation of phosphoinositide 3-kinase (PI3K) and Akt via epidermal growth factor receptor (EGFR) gene amplifications and mutations as well as loss of phosphatase and tensin homolog (PTEN) [12]. Activation of hypoxia inducible factor-1α (HIF-1α) in hypoxic and necrotic tumor regions additionally increases aerobic glycolysis [13].

**Figure 1:**
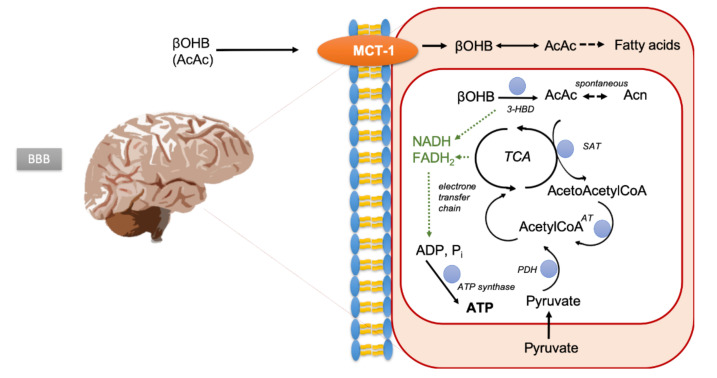
Brain uptake and metabolism of ketone bodies. 3-HBD, hydroxybutyrate dehydrogenase; SAT, succinylCoA:acetoacetate CoA transferase; PDH, pyruvate dehydrogenase; TCA, tricarboxylic acid.

While normal neurons and glial cells can metabolize either ketone bodies (KB; β-hydroxybutyrate (βOHB), acetoacetate (AcAc), and acetone (Ac) formed from acetoacetate by spontaneous decarboxylation) or glucose (Figure 1), it has been suggested that malignant brain tumor cells lack this metabolic flexibility due to a deficiency of enzymes to oxidize ketone bodies [14,15,16,17,18]. KB are formed in the liver via acetyl-CoA predominantly from fatty acids, and blood concentrations increase during fasting or high-fat diets. They can be detected intracerebrally using proton (^1^H) MR-spectroscopic imaging (MRSI) [19,20,21].

The ketogenic diet (KD) is a well-tolerated, high-fat low-carbohydrate diet that can lower circulating glucose and insulin levels when calorie restricted [22,23]. If tumor cells indeed lacked the flexibility to produce adenosine triphosphate (ATP) from ketone body oxidation, KD could result in a selective vulnerability of glioma cells to glucose reduction [24]. In support of this hypothesis, some preclinical studies found that calorie restricted KD (crKD) can reduce tumor growth through effects on angiogenesis, apoptosis, and inflammation and can improve prognosis in high-grade glioma mouse models [25,26,27]. In addition, fasting mimicking diets (FMD) and fasting itself showed an increase in resistance to chemotherapy in normal but not cancer cells of several preclinical cancer models, which could reduce potentially life-threatening side effects of treatments [28,29,30,31].

Another important and energy dependent aspect of tumor metabolism is pH homeostasis. Through changes in the expression of cellular membrane ion transport channels and the CO_2_/HCO_3_^−^ buffering system, tumor cells maintain an ATP-dependent reversed pH gradient with a slightly alkaline intracellular pH (pH_i_) and an acidic extracellular pH (pH_e_), promoting proliferation, migration and invasion [32,33,34]. ATP levels and pH_i_ values can be measured non-invasively in vitro using phosphorous (^31^P) MRSI [35].

The ERGO2 trial (Ernaehrungsumstellung bei Patienten mit Rezidiv eines Glioblastoms) is the first prospective randomized study designed to test feasibility and efficacy of the combination of crKD and radiotherapy in any tumor type. Here, we present results of the MRSI subtrial.

## 2. Results

### 2.1. Patient Characteristics and Prior Treatment MRSI Substudy

**Figure 2:**
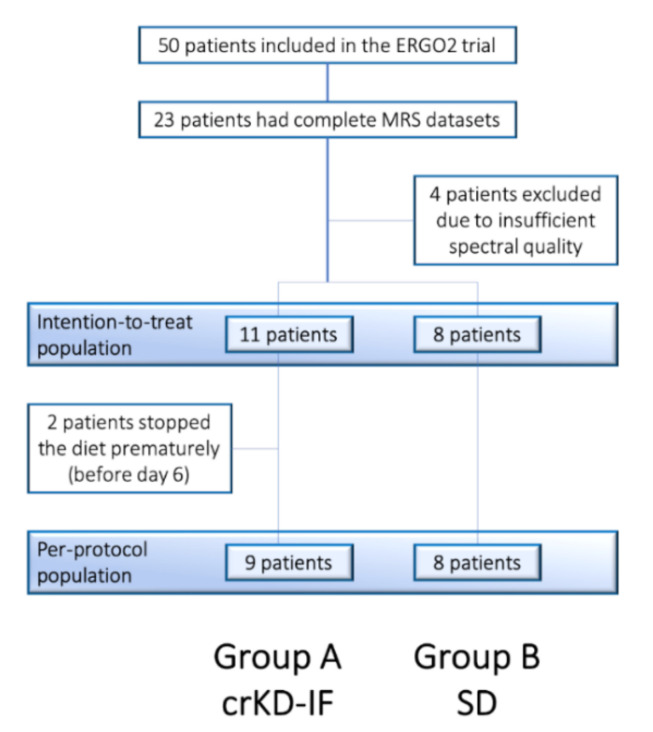
Consort flow diagram of the ERGO 2 MRSI subtrial.

Of 23 patients with complete MRS datasets, four were excluded from the analysis due insufficient spectral quality, leaving 11 patients in Group A with crKD-IF and 8 patients in Group B with SD. These 19 patients defined the intention-to-treat population (ITT; Figure 2).

Patient characteristics and treatment are summarized in Table 1.

Median age at study inclusion was 51 years (Group A: 53 years, Group B: 42.5 years; mean Group A: 53 ± 7, Group B 41 ± 14). 79% of all patients were included with a primary diagnosis of GBM (*n* = 15), while 21% suffered from other higher-grade tumors progressed from lower-grade glioma (*n* = 4). 9% (*n* = 1) of patients in Group A and 50% (*n* = 4) of patients in Group B were surgically resected or re-resected within one month prior to start of study re-irradiation therapy. All of these patients included in the MRS study had only received partial resection for recurrent GBM or suffered from multifocal GBM with non-resected tumor locations.

### 2.2. Adherence to Radiation Study Protocol

Sixteen patients underwent hypofractionated radiotherapy with a total dose of 20 Gy over five consecutive days on day 4–8. Three patients were treated with alternative schemes (Group A: *n* = 1, Group B *n* = 2).

### 2.3. Patients’ Adherence to Recommended Dietary Intervention

Overall dietary requirements were well tolerated in both groups. 2/19 patients (Group A: *n* = 2, Group B *n* = 0) patients did not complete the required nine days of dietary intervention but dropped out after two and four days respectively. The remaining 17 patients defined the per protocol population (PPP).

### 2.4. Detectability of Ketone Bodies in Urine and Blood Samples

At baseline, urine and blood ketone levels as well as fasting blood glucose levels did not differ between the groups. 64% of patients in Group A (*n* = 7) achieved blood ketone levels (βOHB) > 0.5 mmol/L at day 6 during crKD-IF. Mean ketone blood levels (βOHB) in the ITT population of Group A were 1.27 ± 1.23 mmol/L and 1.4 ± 1.23 mmol/L in the PPP. None of the patients in Group B at day 6 during a balanced nutrition achieved a blood ketone level > 0.5 mmol/L, with mean levels of 0.13 ± 0.1 mmol/L. There was a significant difference at day 6 between Group A and B with respect to ketone blood levels (ITT *p* < 0.01, PPP *p* = 0.02; one patient in Group A with no ketone blood levels reported). Five patients in Group A tested positive for urine ketosis (AcAc, Acn) with a median of +++ (range 1.5–16 mmol/L; + = 1.5 mmol/L, ++ 4.0 mmol/L, +++ 8.0 mmol/L, ++++ 16 mmol/L), while one patient was tested negative (four patients with no urine samples obtained). In Group B on day 6 during a balanced nutrition, no patients tested positive for urine ketosis. Within the MRSI substudy in the ITT population, fasting glucose levels in patients of Group A declined during the intervention with mean glucose levels at baseline of 86.4 ± 13 mg/dL and 79.8 ± 14.3 mg/dL on day 6. Comparison of means showed a decline of −6.6 ± 5.6 mg/dL but did not reach statistical significance (*p* = 0.25). With regard to the PPP, similar results could be shown with a decline of −7.4 ± 6.7 mg/dL that did not reach statistical significance (*p* = 0.28).

### 2.5. Intracerebral Detectability and Quantitation of Ketone Bodies Using MRSI

In 4/11 patients at day 6 during crKD-IF (Group A), intracerebral KB were detected with an estimated CRLB < 35% as given by LCModel. All patients displayed an Acn signal at 2.22 ppm. In one of these patients (Patient 26), an Acn signal was fitted in voxels of tumor tissue and normal appearing white matter (NAWM), in all other patients (Patient 27, 34, 38) in voxels of NAWM only. Acn signals were quantified and are listed in Table 2 with respective urine and blood ketone levels.

**Figure 3:**
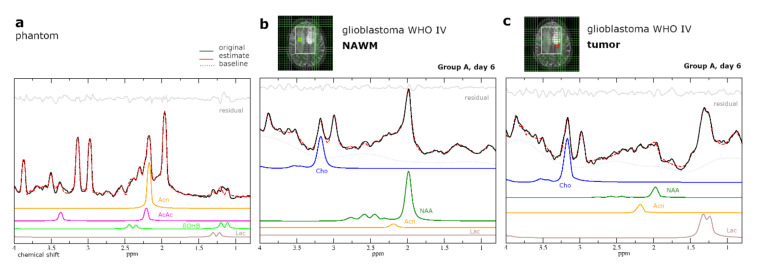
(**a**) Simulated dataset tested on phantom data containing βOHB 1 mM, Acn 1 mM, AcAc 1 mM, Lac 2 mM, and standard metabolites at expected in vivo concentrations. (**b**,**c**) Analysis of patient proton MRSI data (both Patient 38) at day 6 during crKD-IF using LCModel with simulated KB and standard metabolites, as well as a build in basis data set for simulation of MM and lipids. For visualization, a line broadening of 3 Hz was applied to all spectra. Green (NAWM) and red (tumor) boxes indicate voxel positioning on T2-weighted imaging (T2WI). The tumor voxel shown (**c**) was excluded from analysis due to insufficient spectral quality, but shown here to demonstrate the difficulty of data evaluation in these heavily pretreated patients with recurrent high-grade glioma and ongoing re-irradiation therapy. The small Acn signal in the spectrum is comparable to the baseline modulations due to other strongly coupled metabolites, lipids, and macromolecules and this difficult to ascertain visually.

The relationship between MRSI detected KB and KB in blood samples and urine samples was weak with *R*-values of −0.22 and 0.44 respectively (Pearson correlation coefficient). No signals for βOHB or AcAc in either patient group and no signal for Acn in Group B were fitted with CRLB < 35%. ^1^H-MRS spectrum of Patient 38 with Acn fit of NAWM at day 6 during crKD-IF is shown in Figure 3b,c.

### 2.6.Intracellular pH and Energy Metabolism

**Figure 4:**
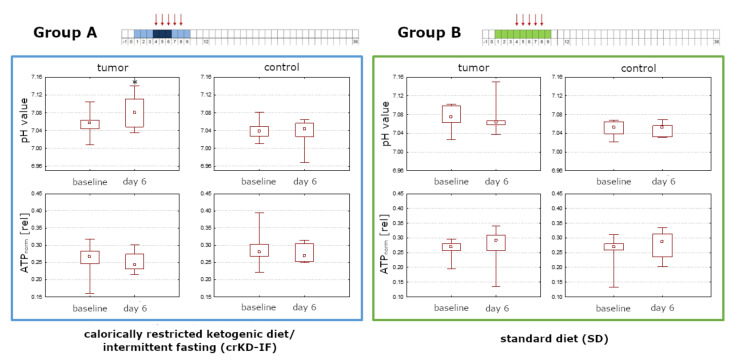
Analysis of the ITT population (11 patients in Group A with crKD-IF and 8 patients in Group B with SD). Group A exhibited a significant increase in pH_i_ in tumor voxels comparing baseline levels to day 6 during crKD-IF (*p* = 0.03), while there were no significant changes reported for Group B (*p* = 0.48). ATP levels were stable in tumor or control voxels of either group comparing baseline to day 6. A Wilcoxon signed-rank test as a paired, non-parametric statistical hypothesis test was used to compare pH_i_ and ATP levels. Box-and-whisker plots with minimum, maximum, interquartile ranges (25th and 75th percentiles) and median are shown.

**Figure 5:**
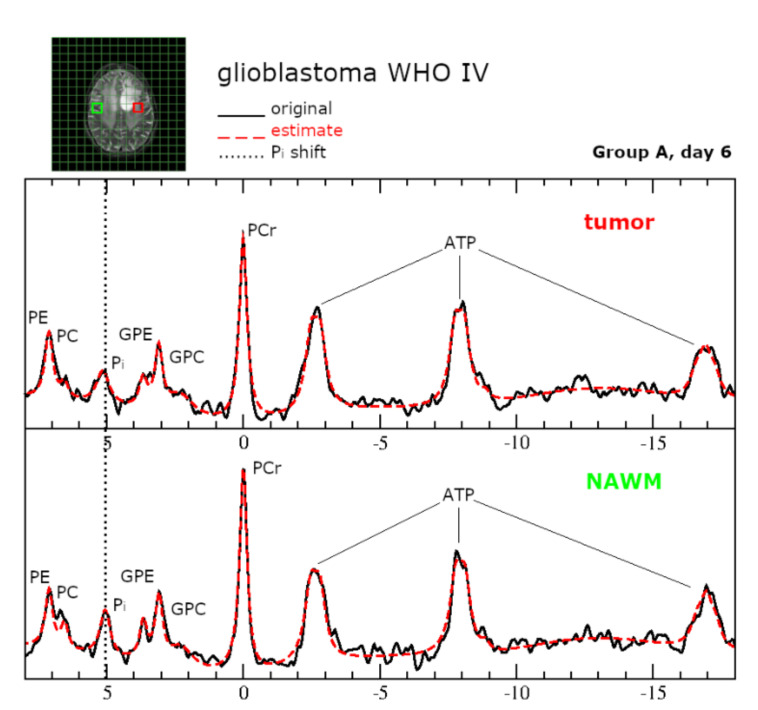
Representative ^31^P spectra for tumor tissue and NAWM at 3T. Green (NAWM) and red (tumor) boxes indicate voxel positioning on T2WI. The original spectrum is depicted as a black line and the spectral fit as a red dotted line. One signal was used to model the inorganic phosphate (P_i_). At physiological pH, H_2_PO_4_, and HPO_4_ ions contribute to the P_i_ signal. Since both components have different chemical shift, the chemical shift (signal position) of inorganic phosphate (P_i_) is pH dependent and can be used as a pH-marker.

For both groups in the ITT population, pH_i_ was significantly lower in control voxels (NAWM) than in tumor voxels (Day 6 Group A: *p* = 0.01; Group B: *p* = 0.02). Group A exhibited a significant increase in pH_i_ in tumor voxels comparing baseline levels to day 6 during crKD-IF (*p* = 0.03), while there were no significant changes reported for Group B (*p* = 0.48). No significant changes in ATP levels were detected in tumor or control voxels of either group comparing baseline to day 6 (Group A tumor: *p* = 0.29; Group A control: *p* = 0.65; Group B tumor: *p* = 0.33; Group B control: *p* = 0.21). Box-and-whisker plots with minimum, maximum, interquartile ranges and median are shown in Figure 4. Representative ^31^P spectra are displayed in Figure 5.

Analyzing the mean values of day 6 tumor ATP and tumor pH_i_ as well as the change of ATP and pH from baseline to day 6, there was no statistically significant difference splitting for serum ketosis (≥0.5 mmol/L or <0.5 mmol/L) or glucose ketone index (>2 mmol/L or ≤2 mmol/L) [36]. Accordingly, there was no statistically significant correlation of ATP or pH_i_ to the serum ketosis or serum glucose at day 6.

Median glucose of all patients was 83.5 mg/dL (4.6 mmol/L) at day 6. An unplanned sub-analysis of the patients of the KD-IF group who completed treatment per protocol had shown, that patients with a glucose below the median had a significantly longer PFS than patients above the median (median PFS 111 days (95%CI 45–178) vs. 42 days (95%CI 41–43), *p* = 0.014) [37]. When applying the same median split (>83.5 mg/dL: *n* = 5, <83.5 mg/dL: *n* = 6) to compare mean values of tumor ATP and pH_i_ at day 6, there was no statistical difference (tumor ATP *p* = 0.98, tumor pH_i_ *p* = 0.79).

## 3. Discussion

### 3.1. Discussion

In our study, Acn was the only reliably MRSI-detectable KB within tumor tissue and/or NAWM. It was only detected in patients during crKD-IF (Group A), not in patients with a balanced diet. But even with elevated ketone serum and urine levels in most patients in Group A, it was merely detected in 4/11 patients.

Reports on MRSI-detectability of KB are not consistent throughout literature and concentrations sometimes fail to show a correlation with serum or urine levels [19,20,38,39]. This may be related to the rather small signal amplitudes of the KB, especially after a short dietary intervention. AcAc and β-OHB as hydrophilic anions can merely cross the blood–brain barrier (BBB) via a monocarboxylate transporter (MCT), which facilitates proton-linked transport of monocarboxylates [40]. Rat studies have shown an upregulation of cerebral MCT-1 after several weeks of ketogenic diet, but not after short periods of fasting (48 h) [41,42]. Acn on the other hand, is both hydrophilic and lipophilic and can be found in aqueous and lipid compartments [43,44]. Its concentration in cerebrospinal fluid is proportional to plasma, indicating free diffusivity across the BBB [45]. Increased overall tissue concentration combined with the high MR sensitivity of the Acn singlet at 2.22 ppm, which arises from six equivalent protons, may account for MR detection of this specific KB while the others remain invisible. The AcAc and βOHB signal originate from three equivalent protons, resulting in half the amplitude for the same molecular concentration compared to Acn. In addition, the 1.2-ppm βOHB signal is a doublet due to J-coupling, so the peak height is further reduced by a factor of 2. Therefore a lower sensitivity of MRSI detection has to be assumed for AcAc and βOHB [20].

MRSI results showed no significant depletion of ATP production in tumor tissue of patients at day 6 during crKD-IF, even though there were a significant difference in ketone blood levels between Group A and B at day 6, and a decline in fasting glucose in Group A from baseline to day 6. The absence of a cellular energy breakdown suggests that tumor cells maintain glycolysis even with reduced glucose levels and in addition may generate ATP through other sources. 

Recently, the hypothesis that brain tumors are metabolically inflexible has been contradicted in vivo (but not in vitro) in two rat glioma models (9L and RG2) [24,46,47,48]. During infusion of ^13^C-labled β-OHB, ^13^C-labeling of glutamate was detected in vivo in tumor voxels using ^13^C/^1^H MR Spectroscopy. Glutamate was labeled through fast exchange with α-ketoglutarate, which is an intermediate metabolite in the tricarboxylic acid cycle and can derive from oxidative metabolism of β-OHB. Contribution of ketone bodies to oxidative metabolism increased when animals were fed a KD with upregulation of MCT-1 in RG2 tumor cells. Histological staining confirmed that tumor cells made up the vast majority of the tissue, demonstrating enzyme capacity of tumor cells to oxidize KB, generating ATP. 

Acidification of the extracellular milieu (low pH_e_) and concomitant intracellular alkalization of the cytoplasm (high pH_i_) are hallmarks of cancer, leading to a reverse pH gradient in cancer cells [49,50]. A rapid increase in intracellular lactate concentration in glycolytic tumors is concomitant with a sharp decrease in pH that starts to relax rapidly towards more alkaline values [51]. Intracellular alkalosis is maintained through several mechanisms including the increased expression and activity of acid extruding plasma membrane transporters, for example, Na^+^/H^+^ exchangers (NHE), H^+^/K^+^-ATPases, and Na^+^/HCO_3_^−^ cotransporters (NBC), which work in concert with carbonic anhydrases (CA) [52]. Since a shift from glycolysis towards oxidative phosphorylation is expected during KD/fasting, less intracellular lactate will be generated in the fasting state. In our study, stable ATP values between baseline and day 6 in Group A were corroborated not only by maintenance, but even a slight increase in alkaline pH_i_ in tumor voxels. Our findings might be the momentary result of an overshoot due to the joint activity of lactate and H^+^ co-transporting MCT1 and MCT4. However, this hypothesis needs to be experimentally confirmed by further studies. Ultimately, low intracellular H^+^ concentrations will favor lactate flux into the cell [52]. The direct influence of ketone bodies on pH_i_ appears less likely. Voronina et al. exposed synaptosomes to high levels of β-OHB and saw no changes in intracellular pH [53]. As opposed to glucose utilization, utilization of ketone bodies does not result in lactate production. Experiments with glioblastoma cell culture further showed that lactate excretion is stable even when β-OHB is available as an additional source of energy [15]. Consistent with these findings, we recently reported that isocitrate dehydrogenase (IDH) mutant tumors, which are less glycolytic, display significantly lower lactate concentrations compared with IDH wild-type tumors and a near-normal pH_i_ [54]. Ultimately in the complex process of cancer metabolism, other factors may come into play—for example a different link of glucose and β-OHB metabolism to redox potential via nicotinamide adenine dinucleotide (NAD) utilization.

### 3.2. Limitations

Due to missing values of patients withdrawing from study or MRS consent, the number of cases available for analysis is reduced and therefore the statistical power. 

In accordance with prior studies, a carefully simulated and constructed LCModel basis set for the specific acquisition and quantitative criteria of KB is necessary. Increased numbers of signal averages with consecutively increased scan time, larger voxels and employment of a semi localization by adiabatic selective refocusing (semi-LASER) sequence may allow improved detection of KB [20]. However, when the protocol for this study was set up in 2013, a semi-LASER sequence was not implemented at our center.

Phantom replacement as an external reference method for metabolite quantitation is affected by several factors that can create variability between measurements in phantom and human subjects such as coil loading, transmit calibration, and receive profile. While the Tofts formula, which was applied in this study, takes some factors such as coil loading into account, others such as the inhomogeneous B1 field obtained at 3 T are not corrected for. Quantitative estimates of metabolite concentrations in patients therefore may differ from their true concentrations. The study protocol did not include the acquisition of water spectra for an internal reference method in addition or as a comparison to the external method.

Unfavorably, ^31^P MRSI has a poor spatial resolution (large voxel sizes) when compared to ^1^H MRSI and an increased spreading of signal into adjacent voxels caused by the point spread function (PSF). The PSF describes the blurring of a point due to coarse k-space sampling. This holds especially true for 8 × 8 × 8 k-space sampling, which is typical for many ^31^P MRSI studies and was employed in this study. The inherent partial volume effect tends to level focal changes in the position of the signal of inorganic phosphate. Since we used only one signal to fit inorganic phosphate, the estimated pH_i_ rather indicates a deviation to higher values compared the real pH_i_ in the target region [55].

## 4. Materials and Methods

### 4.1. Patients and Study Design

ERGO 2 was a randomized, open-label study (ClinicalTrials.gov NCT01754350; DRKS-ID: DRKS00010749), including an extended MRSI protocol that was approved by Ethic Committees of all participating centers (University Hospital Frankfurt am Main, University Hospital Tübingen, University Hospital Erfurt, approval code: 1/13). MRSI was performed at the Frankfurt site only. Study population consisted of patients with recurrence of a histologically confirmed GBM, gliosarcoma or malignant progression of a lesser grade brain tumor in MRI follow-up and a multidisciplinary tumor board (MDT) recommendation for re-irradiation therapy. The initial radiotherapy and the initial surgical resection/biopsy had to be completed at least 6 months prior and a sufficient performance status (Karnofsky-index ≥ 60%) was required. Primary endpoint of the study was progression free survival at 6 months (PFS-6) which has been reported separately [37]. Secondary endpoints addressed in this report were detection and monitoring of cerebral and specifically intratumoral concentrations of βOHB, Ac and AcAc, as well as the impact of crKD-IF on intratumoral ATP levels and maintenance of a reversed pH gradient. 

**Figure 6:**
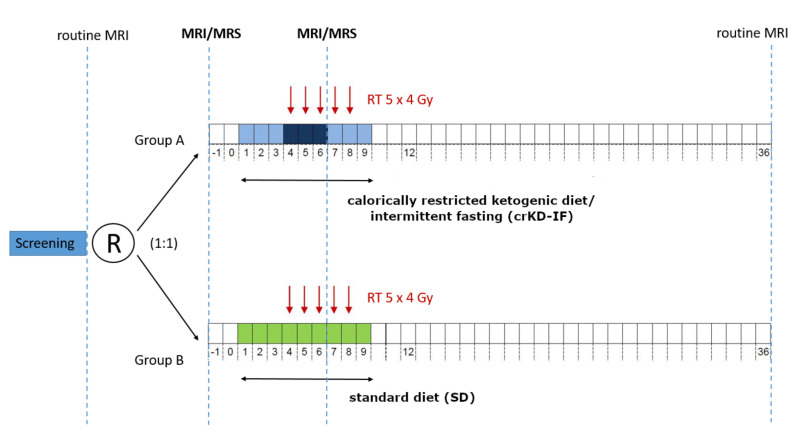
50 patients with recurrent GBM and indication for re-irradiation therapy were randomized (1:1) in two groups: Group A keeping a ketogenic diet with calorie restriction (21–23 kcal/kg/day) for nine days, including three days of fasting (0 kcal/day) and group B keeping a balanced diet. RT was performed on day 4–8 (5 × 4 Gy). 32 received an extended MRS examination at baseline (day −1) and 23/32 on day 6. MRSI examinations were scheduled in addition to routine follow-up. Adapted from Voss et al. [37].

The 50 patients enrolled at all centers were randomized 1:1 in two groups: Group A keeping a crKD for nine days, including three days of fasting and group B keeping a balanced diet over the same period. For Group A, crKD was scheduled from day 1–3 (21–23 kcal/kg/day, max. 50 g carbohydrates/day), and again from day 7–9 (21–23 kcal/kg/day, max. 50 g carbohydrates/day). During days 4–6, fasting (0 kcal/day) was required with unlimited supply of liquids, at least 1.5–2.0 L/day (water, tea, vegetables broth). For Group B, a complete and balanced nutrition as recommended by the German Nutrition Society (DGE) with approximately 60–80 g fat, 5 g/kg bodyweight carbohydrates and 0.8 g/kg bodyweight proteins were ensured. All patients were counseled by a DGE-certified nutritionist who developed a patient specific diet plan and provided ketogenic recipes to patients in Group A. Body weight was closely monitored and diet was terminated in case of a weight loss of more than 10% of the initial body weight, severe and symptomatic hypoglycemia, or any common toxicity criteria (CTC) grade 4 adverse event related to the diet. Hypofractionated radiotherapy was performed after three-dimensional CT planning with a total dose of 20 Gy over five consecutive days on day 4–8 (5 × 4 Gy) [56]. Further clinical follow-up visits were scheduled at day 12, after one month and hereafter every two months. Study design is outlined in Figure 6.

### 4.2. Magnetic Resonance Imaging

Of the 50 patients enrolled at all centers, 32 received ^1^H/^31^P MRSI at baseline (day −1) and 23/32 on day 6. Day 6 corresponds to the third day of fasting after three days of crKD (21–23 kcal/kg/day) in the intervention group and the third day of radiotherapy (5 × 4 Gy). MRSI was scheduled in addition to routine follow-up scans and was declined by a number of patients. Examinations were performed on a clinical whole-body 3 T MR Scanner (Magnetom Trio, Siemens Medical Solutions, Erlangen, Germany), using a double-tuned ^1^H/^31^P volume head coil (Rapid Biomedical, Rimpar, Germany). The MR protocol included 3D T1-w gradient echo sequences, T2 weighted turbo spin echo, ^1^H decoupled ^31^P MRSI with 3D CSI recording the FID, as well as with ^1^H MRSI recording the spin echo at TE 30 ms. Tumor tissue was identified on previously acquired standard-MRI images with gadolinium-based contrast agent in tumors with disruption of the blood–brain barrier and on newly acquired T2 weighted images. For ^1^H MRSI the volume of interest (VOI) was selected by a combination of the point resolved selected spectroscopy (PRESS) and outer volume suppression, aimed to enclose the recurrent tumor as well as contralateral NAWM. Details on MRSI protocol are listed in Table 3.

### 4.3. Data Analysis

Registration of the acquired multimodal spectroscopic data to 3D-anatomical data was performed with an in-house software tool scripted in MATLAB (The Mathworks, Inc., Natick, MA, USA) [57]. The tool provides a graphical user interface which facilitates the selection of voxels from the entire 3D spectroscopic data set using 3D-T1 weighted and T2 weighted reference images with an MRSI grid overlay. Voxels were selected from the area of the recurrent tumor. Control voxels were selected in areas of NAWM on the contralateral hemisphere, anatomically corresponding to the selected tumor area. Spectra of insufficient quality (linewidth of the water signal (FWHM) > 0.1 ppm, Cramér–Rao lower bounds (CRLB) of choline as given by LCModel > 15%, large artifacts), e.g., those close to the skull/brain interface, were excluded from the analysis. Considering the expected low concentrations of cerebral KB in our study design, a rejection threshold using CRLB of a metabolite fitting of 35% was applied to Acn, AcAc, and βOHB. Above this threshold, the metabolite was considered as undetectable [58].

Analysis of patient proton MRSI data was performed in the frequency domain using LCModel (version 6.3-1C), a non-iterative commercially available software package that analyzes in vivo MRS data as a linear combination of model spectra which were simulated, and a build in basis data set for simulation of MM and lipids [59]. For each selected proton voxel, corresponding phosphorous data were analyzed with the software package jMRUI (Version 6.0 beta) [60] employing a non-linear least square fitting algorithm (AMARES) in the time domain [61].

### 4.4. ^31^P MRSI Model

The model was composed of 14 exponentially decaying sinusoids. The signal of phosphocreatine (PCr) was adjusted to 0 ppm, and constraints for phosphocholine (PC), phosphoethanolamine (PE), glyceroethanolamine (GPE), and glycerophosphocholine (GPC) signals were applied keeping the chemical shifts at a fixed difference with regard to the position of PCr, while adjusting the linewidth to the value of PCr. ATP was modeled by 7 sinusoids assuming two doublets and a triplet with an 18 Hz coupling constant. One signal with a fixed chemical shift of 2.24 ppm and maximum line width of 50 Hz was used to account for potential macromolecule signals in the phosphodiester region [62,63]. One signal was used to model the inorganic phosphate (P_i_) in the spectra region between 3.3 and 5.0 ppm. At physiological pH, H_2_PO_4_ and HPO_4_ ions contribute to the P_i_ signal. Since both components have different chemical shift, the chemical shift (signal position) of inorganic phosphate (P_i_) is pH dependent and can be used as a pH-marker [55]. Referring to the approach of Petroff et al. [64] (i.e., calculating pH according to pH = pkA + 10log (δ1 − δ0/δ0 − δ2)), the pH values of the predefined areas of interest were determined from the chemical shift difference between P_i_ and PCr. The formula is implemented in jMRUI with the following default values: pkA 6.75 ppm, delta1 3.27 ppm, delta2 5.63 ppm. Fitting the ^31^P spectra, all signals were generally assigned correctly as visually assessed, leaving a flat line for the residual with equal distribution of noise. Changes in ATP concentrations were monitored using the intensity of ATPß signal, normalized to the intensity of the other phosphate containing metabolites (ATP ß/(PEth + GPE + GPC + PCre + ATPß)). Each metabolite symbol represents its intensity as given by jMRUI AMARES. The normalization corrects for variations in coil sensitivity due to different coil loadings, while still being sensitive to changes in ATP concentrations.

### 4.5. Simulation of Basis Set for ^1^H MRSI

The basis set for LCModel was simulated using the NMRScope-B plugin, which is implemented in jMRUI (Version 6.0 beta) [65]. MR spectra for each metabolite were calculated based on a priori knowledge of scalar coupling, chemical shifts, and vendor specific hardware parameters for data acquisition. The metabolites simulated and included in the data evaluation were: sodium-3-hydroxybutyrate (βOHB), acetone natural (Acn), lithium acetoacetate (AcAc), sodium l-lactate (Lac), *N*-acetyl-l-aspartic acid (NAA), creatine anhydrous (Cr), choline chloride (Cho), l-glutamine (Gln), l-glutamic acid (Glu), and myo-inositol (mI) (all Merck KGaA, Darmstadt, Germany). A line broadening of 3 Hz was applied to each basis spectrum.

Following simulation, the basis set was tested on phantom data, including signals for the respective metabolites at expected in vivo concentrations (Lac 2 mmol/L, NAA 8 mmol/L, Cr 6 mmol/L, Cho 2 mmol/L, Gln 2 mmol/L, Glu 7 mmol/L, mI 4 mmol/L). Concentrations of βOHB, Acn and AcAc were at 1 mM. The frequency, phases and linewidths of the peaks were all constrained relative to the creatine singlet at 3.03 ppm (Figure 3a).

### 4.6. Phantom Replacement

A spherical phantom was prepared containing 2600 mL of an aqueous solution (Dulbecco’s phosphate buffered saline, DPBS) and the respective metabolites at physiological pH (pH adjusted to 6.96 by titration with NaOH). The spherical size was chosen to minimize susceptibility artifacts. The phantom was placed in the center of the dual tuned head coil. Measurements lead to well-resolved spectra with flat baselines. Using the phantom replacement technique as described by Tofts et al. and applied by our group before, metabolite levels for Acn were calculated [66,67]. Data were acquired with a transmit/receive coil. Regarding the principle-of-reprocity we applied corrections as described by Michaelis et al., which are also included in the Tofts formula [68]. Relaxation effects for Acn signals at 3T (signal loss) were corrected assuming previously published values for NAA for T2 and T1 (T2 = 270 ms; T1 = 1.4 s, correction factor 0.59) [69,70].

### 4.7. Laboratory Testing

Patient urine samples were analyzed using Combur^®^ 10 Test Strips (Hoffmann-La Roche, Basel, Switzerland), while capillary blood samples were analyzed using the Precision Xceed system with blood β-ketone test strips (Abott, Chicago, IL, USA). Combur^®^ Test Strips detect AcAc (detection limit approximately 0.5 mmol/L) and Acn (detection limit approximately 7 mmol/L), while blood test strips detect βOHB (detection limit < 1 mmol/L).

### 4.8. Statistics

Statistical analysis was performed using a commercially available software (STATISTICA, version 7.1; StatSoft, Tulsa, OK, USA). A Wilcoxon signed-rank test as a paired, non-parametric statistical hypothesis test was used to compare pH_i_ and ATP levels at baseline and on day 6 (equal to the third day of fasting after three days of crKD) within tumor tissue and NAWM. The same test was used to compare pH_i_ and ATP levels within tumor tissue and NAWM at each point of time. An unpaired *t*-test was used to compare blood ketone levels of Group A and B and a paired *t*-test to compare glucose levels at baseline and on day 6 for each group. For all tests, *p* < 0.05 was considered significant. The Pearson correlation coefficient was used to measure the strength of a linear association between MRSI detected KB and KB in urine and blood samples.

## 5. Conclusions

MRSI allowed specific detection of ketosis in patients with recurrent GBM during crKD-IF, but sensitivity was low. The intervention did not affect tumor ATP levels or pH_i_ the way, that would reflect a selective vulnerability of tumor cells to glucose starvation. According to our findings, tumor cells maintain energy homeostasis even with reduced glucose levels through either enhanced uptake of glucose or generation of ATP through other sources, possibly even KB oxidation. Findings of the subtrial are in line with the negative results of the main trial [37] with regard to progression free and overall survival (PFS und OS) and suggest that metabolic interventions targeting brain tumor metabolism need to be further refined.

## Figures and Tables

**Table 1:** Patient characteristics MRS substudy. Decimal round method if not otherwise specified.

Characteristics	All Patients (*n* = 19)	Group B (*n* = 8)
	Group A (*n* = 11)	
**General**		
Age, median	53 (range 38–64)	42.5 (range 25–61)
Age, mean	53 (SD = 7)	41 (SD = 14)
Karnofsky index, mean rounded to nearest tenth (SD)	90 (SD = 13)	90 (SD = 6)
**Histology**		
Glioblastoma, IDH wildtype, WHO IV (*n*)	5	3
Glioblastoma IDH-mutant, WHO IV (*n*)	1	1
Glioblastoma IDH-mutant, IDH unknown, WHO IV (*n*)	4	1
Anaplastic astrocytoma, IDH wildtype, WHO III (*n*)	1	0
Oligodendroglioma, IDH-mutant and 1p/19q co-deleted, WHO II (*n*)	0	1
Pleomorphic Xanthoastrocytoma, IDH wildtype (*n*)	0	1
Undifferentiated neuroepithelial tumor, NOS, WHO IV (*n*)	0	1
**MGMT promotor methylation status**		
Not methylated	46% (*n* = 5)	63% (*n* = 5)
Methylated	46% (*n* = 5)	25% (*n* = 2)
Not known	9% (*n* = 1)	13% (*n* = 1)
**Prior treatment**		
Radiation therapy up to 60 Gy	100% (*n* = 11)	100% (*n* = 8)
Concomitant TMZ	100% (*n* = 11)	88% (*n* = 7)
Median of adjuvant cycles of TMZ	6	3
Prior treatment with Bevacizumab	18% (*n* = 2)	13% (*n* = 1)
Resection prior to study treatment	9% (*n* = 1)	50% (*n* = 4)
**Time interval first and second irradiation (months)**		
Median	9	11
Mean	14 (SD = 9)	17 (SD = 16)

**Table 2:** Quantitation of intracerebral KB in patients with KB signals with an estimated CRLB < 35% as given by LCModel

Patient ID	Group	Drop out during 9 Days of crKD-IF	Histology at First Diagnosis	Glucose Baseline (mg/dL)	Glucose Day 6 (mg/dL)	Blood Ketone Levels Day 6 (mmol/L)	Urine Ketosis Day 6 (mmol/L)	Acn Signal Quantitation (mmol/L)
26	A	yes	Glioblastoma	78.0	59.0	0.1	0	tumor tissue 0.12; NAWM 0.14
27	A	yes	Glioblastoma	84.0	98.0	N/A	N/A	NAWM 0.28
34	A	no	Anaplastic astrocytoma	112.0	78.0	0.4	1.5	NAWM 0.21
38	A	no	Glioblastoma	81.0	54.0	4.5	N/A	NAWM 0.25

**Table 3:** MR-spectroscopy sequence protocol. TR, repetition time, TE, echo time, FID, free induction decay, CSI, chemical shift imaging, PRESS, point resolved spectroscopy, * nominal size due to k-space sampling, ** defined by slice selection, *** delay between excitation and data acquisition.

Sequence	Slice Thickness	TR; Excitation Flip Angle	TE	Matrix Size; in Plane Resolution	Vector Size; Bandwidth	Scan Time
3D FID ^31^P CSI	25 mm *	2000 ms; 60°	2.3 *** ms	8 × 8 × 8 at 240 × 240 × 200 mm^3^ FOV interpolated to 16 × 16 × 16; 30 × 30 mm^2^ *	1024; 2000 Hz	10:44 m
2D ^1^H PRESS sequence	12.5 mm **	1500 ms; 90°	30 ms	16 × 16 at 240 × 240, interpolated to 32 × 32; 15 × 15 mm^2^ *	1024; 2000 Hz	4:45 m
